# Management of circulatory failure after Fontan surgery

**DOI:** 10.3389/fped.2022.1020984

**Published:** 2022-11-08

**Authors:** Alicia M. Kamsheh, Matthew J. O’Connor, Joseph W. Rossano

**Affiliations:** Division of Cardiology, Children’s Hospital of Philadelphia, United States

**Keywords:** Fontan, heart failure, single ventricle, medications, ventricular assist device, Fontan failure

## Abstract

With improvement in survival after Fontan surgery resulting in an increasing number of older survivors, there are more patients with a Fontan circulation experiencing circulatory failure each year. Fontan circulatory failure may have a number of underlying etiologies. Once Fontan failure manifests, prognosis is poor, with patient freedom from death or transplant at 10 years of only about 40%. Medical treatments used include traditional heart failure medications such as renin-angiotensin-aldosterone system blockers and beta-blockers, diuretics for symptomatic management, antiarrhythmics for rhythm control, and phosphodiesterase-5 inhibitors to decrease PVR and improve preload. These oral medical therapies are typically not very effective and have little data demonstrating benefit; if there are no surgical or catheter-based interventions to improve the Fontan circulation, patients with severe symptoms often require inotropic medications or mechanical circulatory support. Mechanical circulatory support benefits patients with ventricular dysfunction but may not be as useful in patients with other forms of Fontan failure. Transplant remains the definitive treatment for circulatory failure after Fontan, but patients with a Fontan circulation face many challenges both before and after transplant. There remains significant room and urgent need for improvement in the management and outcomes of patients with circulatory failure after Fontan surgery.

## Introduction

The Fontan circulation is a palliative circulation for single ventricle congenital heart disease (CHD) characterized by the lack of a subpulmonary ventricular pump with passive pulmonary blood flow driven by elevated central venous pressures. Since Francis Fontan first reported the successful treatment of two patients with tricuspid atresia in 1971, there have been multiple advances in surgical technique and postoperative management which have led to improvement in short- and long-term mortality in this patient population. Operative mortality is now close to 1% and transplant-free survival at 5 and 10 years is 95% and 90%, respectively (1–4). Today, patients have an estimated 30-year survival after Fontan of approximately 85% (1). Given the improving mortality, there is now a growing number of patients with a Fontan circulation worldwide, estimated at up to 70,000 in 2018 and expected to double over the next 20 years (1, 5). The average age of patients with a Fontan circulation is also expected to increase over the coming years, which will only add to the growing burden of morbidity among this population. These patients are at risk for the multitude of morbidities associated with this unique circulation. This review aims to describe the epidemiology and common modes of circulatory failure in patients with a Fontan circulation and examine management strategies utilized in this population.

## Fontan circulatory failure epidemiology

The morbidities seen in patients with a Fontan circulation are a consequence of the baseline hemodynamic abnormalities associated the Fontan. These include elevated central venous pressure, chronically low preload to a single ventricle leading to decreased cardiac output, and impaired ability to augment cardiac output to meet metabolic demands. Some clinicians have claimed that since patients with a Fontan circulation are unable to augment their cardiac output to meet demands of exercise at low filling pressures, all patients with a Fontan have a chronic form of heart failure (1). There are a number of different definitions of heart failure in the literature, but a recent international consensus definition was proposed as the following: “a clinical syndrome with symptoms and/or signs caused by a structural and/or functional cardiac abnormality and corroborated by elevated natriuretic peptide levels and/or objective evidence of pulmonary or systemic congestion” (6–8). There is overlap between this definition and Fontan circulatory failure, for which the consensus definition is “a broad, non-specific term describing dysfunction of the Fontan circulation that affects a patient’s ability to carry out daily life activities (9).” In this review, we will include causes of heart failure under the broad array of possible underlying etiologies of Fontan circulatory failure (Table 1) (1, 9–14). There does, however, remain difficulties with interchangeability and ambiguity in use of these terms in the literature.

**Table 1:** Common etiologies of Fontan circulatory failure.

• Systolic dysfunction • Diastolic dysfunction • Atrioventricular valve regurgitation • Lymphatic Insufficiency • Arrhythmia • Anatomic obstruction

Symptomatic circulatory failure is common in patients with a Fontan. One single center cohort of patients with a Fontan circulation followed over an average of 15.7 years found that 40% exhibit clinical heart failure (10). In another cohort of adult patients with a Fontan circulation, freedom from Fontan failure was 91% at 10 years, 79% at 20 years, and 59% at 25 years after Fontan operation (15). Among all adults with CHD in the Dutch CONCOR registry, adults with single ventricle heart disease had the highest rate of admission for heart failure, with a hazard ratio of 11.4 when compared to patients with ventricular septal defect. This was more than double the hazard ratio calculated for patients with congenitally corrected transposition of the of the great arteries (16). Identified risk factors for Fontan failure include hypoplastic left heart syndrome, greater age at Fontan surgery, longer length of stay at the time of Fontan surgery, progressive systolic and diastolic ventricular dysfunction, atrioventricular valve regurgitation, sustained arrhythmia, and weight gain in adulthood (15, 17–20).

Given the high-risk of circulatory failure in this population, the American Heart Association has recommended an intensive surveillance strategy in adolescents and adults with a Fontan circulation (1). This includes obtaining an exercise stress test every 1–3 years, cardiac MRI every 2–3 years and a surveillance catheterization every 10 years. This may help to identify patients with worsening hemodynamics before they demonstrate symptoms. Once a patient with a Fontan circulation demonstrates clinical heart failure, the prognosis is quite poor (15, 16, 21–23). In a multicenter U.S. study of patients with CHD admitted for heart failure, 28% of single ventricle patients died during admission (21). Another study of patients with a Fontan found that once Fontan failure was manifest, freedom from death or transplant was 70% at 5 years and only 40% at 10 years (15). This prognosis is considerably worse than patients with a Fontan circulation without clinical symptoms, with signs and symptoms of heart failure conferring a 6.3-fold increased risk of death or transplant (22). Predictors of death or transplant that have been identified in the literature include older age, atriopulmonary or atrioventricular type Fontan, morphologic right ventricle, clinically relevant arrhythmia, low heart rate reserve, lower resting oxygen saturation, protein-losing enteropathy, elevated BNP, poorer ventricular performance, poorer functional health status, lower peak VO2, decline in percent predicted VO2 by 3 or more percentage points per year, and higher atrial pressure (2, 22–25).

## Modes of Fontan circulatory failure

### Systolic dysfunction

Systolic ventricular function is typically normal at the time of Fontan but worsens over time (15, 23, 26). In one large cohort of Fontan patients followed for over 20 years, it was found that Fontan failure occurred at a median of 18.1 years after Fontan completion and 46% of these patients had systolic dysfunction. Fontan failure was more commonly associated with a decline in ventricular function than increasing pulmonary vascular resistance (15). About 50% of patients with a Fontan circulation that are listed for transplant have ventricular dysfunction (27–29).

The underlying cause of the decline in systolic function over time in patients with a Fontan circulation is not clear. During the earlier phases of staged palliation, the ventricle is volume loaded and exposed to chronic hypoxemia, which may predispose to dilation and later dysfunction, although studies have demonstrated that ventricular size and hypertrophy improve initially following the Fontan operation (1, 30). Hypoplastic left heart syndrome (HLHS) confers higher risk of systolic dysfunction because the right ventricular morphology is not embryologically or structurally designed to pump against systemic afterload (10, 26). Myocardial fibrosis has also been seen in this population and is associated with lower mean ejection fraction (31). Fibrosis and abnormal myofiber orientation may predispose to mechanical dyssynchrony over time, which has been demonstrated to be associated with reduced systolic function (32–34). Further, the Fontan circulation is a chronically preload deprived state, which may lead to decreased contractility due to chronic lack of fiber stretch and increased muscle stiffness (1).

Patients with a Fontan circulation are particularly affected by systolic dysfunction due to their baseline low cardiac output state. Additionally, preload is further reduced by loss of the normal suction force which occurs with descent of the atrioventricular valve towards the apex of the heart in systole. This force drives blood through the pulmonary vasculature in the normal circulation. The effect of this force becomes relatively more important in patients with a Fontan circulation who do not have a subpulmonary ventricle but is lost in patients with significant systolic dysfunction (35). There has additionally been recent interest in whether atrial function predicts exercise parameters and Fontan outcomes, although atrial function has not been comprehensively studied and the published data is mixed (36–38).

While systolic dysfunction is typically diagnosed by echocardiogram, there are challenges in using echocardiography to assess systolic function in patients with a Fontan circulation, particularly in patients with a systemic right ventricle (39, 40). There is evidence that utilization of cardiac MRI improves risk stratification in patients with a Fontan circulation and thus many centers will use a combination of echocardiography and cardiac MRI to monitor patients for systolic dysfunction over time (40). In a large study of patients with a Fontan circulation who underwent cardiac MRI, lower end-diastolic volume was the strongest predictor of transplant-free survival. For patients with ventricular dilation (end diastolic volume ≥156 ml/BSA), worse global circumferential strain was particularly predictive of death or transplant (41). On multivariable analysis, end-diastolic volume, end-systolic volume, global circumferential strain, and ventriculoarterial coupling ratio by MRI have been shown to be independently predictive of death or transplant. These measurements are thought to be more useful than ejection fraction because ejection fraction does not account for ventricular dilation. Cardiac MRI can also identify cardiac fibrosis which may contribute to dilatation and dysfunction, although additional work is needed to further elucidate the role cardiac fibrosis may have in this process (42). Further, stress MR to identify patients with decreased ability to augment their ejection fraction in response to dobutamine-stress may also help to identify patients at risk of adverse outcomes (43).

### Diastolic dysfunction

Diastolic dysfunction is increasingly being recognized in the Fontan population, affecting approximately 70% of patients, including >80% of patients with a systemic right ventricle (1, 12, 26, 34, 44–46). The single ventricle has multiple possible underlying etiologies for diastolic dysfunction, including dyssynchrony, myocardial scarring, abnormal geometry, increased wall stress, hypoxemia and history of volume loading prior to the Fontan (1, 45). There is evidence of early relaxation abnormalities leading to prolongation of isovolumetric relaxation which reduces early rapid filling and leads to abnormal wall motion and flow (34, 44). This abnormality is then later compounded by a slow reduction in ventricular compliance which also affects late diastolic filling (12, 44).

Increased filling pressures are not well tolerated by patients with a Fontan due to the requirement for passive pulmonary blood flow. Higher filling pressures lead to higher atrial pressures and a lower gradient for forward flow through the pulmonary vasculature. This leads to decreased preload and ultimately decreased stroke volume even without changes in systolic function (45).

Despite the high prevalence of diastolic dysfunction is in this population, it can be difficult to detect, particularly in early stages. Standard echocardiographic Doppler and tissue Doppler parameters are frequently abnormal but have been shown to correlate poorly with clinical outcomes and only modestly with catheterization measurements (46, 47). Cardiac catheterization is considered the standard for measurement of diastolic filling pressures but is invasive and can miss patients with early-stage diastolic dysfunction because it only measures filling pressures in the resting state (45). Speckle tracking echocardiogram has shown some promise in evaluation of diastolic function, but further work is needed to validate this method in single-ventricle patients (48).

### Atrioventricular valve regurgitation

Atrioventricular (AV) valve failure, defined as requiring valve repair or replacement or development of moderate or greater AV valve regurgitation (AVVR), is seen in approximately 7% of patients with a Fontan circulation at 5 years, 12% at 10 years and 21% at 20 years (20). AVVR in patients with a Fontan circulation may be secondary to annular dilation, leaflet dysplasia, leaflet tethering, leaflet prolapse, or abnormal subvalvar apparatus (49–51). Patients with hypoplastic left heart syndrome are at particular risk for developing AV valve failure, as systemic right ventricular morphology has been found to be an independent risk factor for AVVR (20, 26). There has also been a demonstrated association between older age at Fontan and more severe AV valve regurgitation (26).

AV valve regurgitation is poorly tolerated in this population as the regurgitation leads to an abnormal feedback loop of dysfunction, worsening dilation and worsening AVVR (50). This further decreases cardiac output and increases atrial pressure, which in turn lead to symptoms of low cardiac output or congestion. Significant AVVR also interferes with interpretation of standard indices of ventricular function, such as ejection fraction, and thus practitioners may underestimate the degree of systolic dysfunction in a single ventricle patient with AVVR (52). Furthermore, AVVR interferes with the aforementioned forward sucking force on the pulmonary venous blood during ventricular systole that becomes relatively more important in single ventricle patients (35). Given this, it is unsurprising that a patient with a Fontan circulation with AV valve failure has been found to have more than double the risk of Fontan failure (20).

Multiple imaging modalities can be utilized in the diagnosis and management of AV valve regurgitation in Fontan patients (53). Though 2D transthoracic echocardiography remains the most common imaging modality used, it is less sensitive to the diagnosis of specific etiologies such as leaflet dysplasia and structural abnormalities. The recent development of 3D echocardiography has addressed some of these limitations, by improving assessment of valve morphology, geometry and subvalvar apparatus. Cardiac MRI can evaluate flow and quantify AVVR but can be limited by artifact in patients with stents or devices. Alternatively, computed tomography can be considered in patients with metallic stents or pacemakers that do not allow for cardiac MRI.

### Lymphatic insufficiency

While all patients with a Fontan circulation have lymphatic congestion secondary to the obligate increase in central venous pressure, a subset of patients will develop leakage of chyle into the bronchial, pleural, peritoneal, or intestinal cavities. This results in plastic bronchitis, chylous effusions, chylous ascities, or protein losing enteropathy (1, 14). It is estimated that protein losing enteropathy (PLE) effects between 4% and 13% of patients after Fontan (23, 54, 55). Plastic bronchitis (PB) effects comparatively fewer patients, approximately 2%–4%, although the burden of subclinical PB is likely significant (56, 57). Several factors likely contribute to which patients develop lymphatic complications and which lymphatic disease they manifest, including systemic venous pressures, anatomic Fontan obstruction, lymphatic vessel anatomic differences, prior interventions on the thoracic duct, concomitant cardiac dysfunction, increased mesenteric vascular resistance, proinflammatory state, and genetic differences (1, 58–64). A classification scheme to describe neck and thoracic lymphatics abnormalities has been developed which has been shown to be associated with adverse surgical outcomes after Fontan including mortality, duration of effusions and duration of hospital stay (62). While not a statistically significant association, possibly due to a small sample size, plastic bronchitis and need for transplant within 3 years only occurred in patients with the most severe type 4 abnormalities.

These conditions, which collectively can be referred to as lymphatic insufficiency, can lead to malnutrition, edema, immune deficiency, chronic cough, respiratory distress, hypoxemia and eventually death, although outcomes have improved in the past decade (14, 54, 65). PLE is generally diagnosed by using alpha-1 antitrypsin clearance in a 24-hour stool collection or an elevated alpha-1 antitrypsin level with serum hypoalbuminemia, symptoms of edema, and no other known cause. Imaging modalities such as lymphoscintigraphy and dynamic contrast magnetic resonance lymphangiography can also be used to support the diagnosis of PLE (64). Routine monitoring of serum albumin concentration in patients with a Fontan circulation can identify subclinical albumin loss which may indicate deteriorating hemodynamics (1). Plastic bronchitis is typically diagnosed clinically by patient expectoration of an airway cast or after bronchoscopic removal.

### Arrhythmia

Arrhythmias are common in patients with a Fontan circulation. Sinus node dysfunction and supraventricular tachycardia are most common, affecting up to 60%–80% of patients with a Fontan, although exact rates vary depending on type of Fontan procedure (1). Contemporary surgical techniques such as extracardiac and lateral tunnel Fontan appear to have lower prevalence of arrhythmia as compared to the atriopulmonary type Fontan, but longer follow up is needed to evaluate these newer techniques (1, 17). Ventricular tachycardia is much less common, affecting 5%–12% of the population (66–68). It is estimated that approximately 10% of patients with a Fontan circulation will require pacemaker implantation (69).

Etiologies of arrhythmia in patients with a Fontan circulation include sinus node injury, disruption of the arterial supply of the sinus node, suture lines interfering with conduction, atrial dilation, hypertrophy leading to elevated pressures, the underlying genetic cause of the congenital heart defect causing altered tissue organization, and fibrosis secondary to injury from cyanosis or bypass resulting in altered conduction (1). Arrhythmias further worsen the limited cardiac reserve in a patient with a Fontan circulation. Patients with arrhythmia are significantly more likely to develop heart failure and in one study were found to have a 6-fold increased hazard of death or transplant (19, 22). Atrioventricular synchrony appears to be particularly important to maintain hemodynamics in patients requiring pacing after a Fontan operation (69).

Given the frequency of arrhythmia in Fontan patients, Holter monitoring is recommended every 1–2 years in adolescents and adults, and should particularly be considered in patients experiencing signs and symptoms of Fontan failure (1).

## Treatment

The treatment of circulatory failure in patients with a Fontan is dependent upon identification of the underlying etiology of the failure (Table 2). However, evidence-based recommendations for treatment are limited, with considerable variability among centers with respect to management (70). Surgical and catheter-based interventions, if indicated, typically have superior outcomes to medical therapy, although outcomes vary depending on intervention and indication. Oral medical therapies are typically not very effective, and if there are no surgical or catheter-based interventions to improve the circulation, patients with severe Fontan failure often require inotropic medications or mechanical circulatory support to support the circulation until transplant.

**Table 2:** Management strategies for Fontan circulatory failure.

Catheter Based Interventions	• Creation of a fenestration • Angioplasty and stent angioplasty • Lymphatic intervention including selective lymphatic embolization, thoracic duct embolization (less preferred), thoracic duct decompression • Catheter ablation
Surgical Interventions	• Fontan takedown • Fontan revision • Fontan conversion • Atrial arrhythmia surgery • Surgical innominate vein reimplantation • Biventricular conversion • Atrioventricular valve repair or replacement
Medical Therapy	• Diuretics • Renin-angiotensin-aldosterone system blockers including ACE-inhibitors, angiotensin II receptor blockers, aldosterone antagonists • β-blockers • Modulators of pulmonary vascular resistance including phosphodiesterase type 5 inhibitors, endothelin receptor antagonists and prostacyclin analogs • PLE directed therapy including midodrine, oral budesonide, octreotide • Antiarrhythmics • Optimizing pacing including AV synchrony and resynchronization therapy • Exercise training
Advanced Heart Failure Therapy	• Inotropes • Mechanical circulatory support • Transplant

Timing of referral to a heart failure specialist may also influence the outcomes of various treatment in these patients. Given concern among pediatric heart failure specialists that patients with a Fontan circulation were being referred too late, when advanced heart failure was already manifest and there was irreversible end-organ damage or clinical instability, the ACTION Network has developed a quality improvement project aimed at improving timely referral to a heart failure specialist through standardized referral guidelines. These guidelines include detailed thresholds of cardiac/systemic ventricular dysfunction, Fontan pathway dysfunction, lymphatic dysfunction, and extra-cardiac dysfunction which the ACTION group feels should prompt considered for referral to an advanced heart failure specialist (71, 72). The quality improvement metric that will be measured is proportion of Fontan patients who do not require escalation of heart failure care within 30 days of referral to the heart failure service and data collection is ongoing.

### Catheter-based interventions

A variety of catheter-based interventions can be used to improve the Fontan circulation. In patients with obstruction within the Fontan pathway or pulmonary arteries, stent angioplasty may address obstruction and improve lymphatic failure (61, 73). Patients without a patent Fontan fenestration with PLE have been shown to benefit from creation of a fenestration, although this intervention comes at the expense of worsening hypoxemia and the risks of systemic embolization of venous thrombosis (54, 65, 74).

Lymphatic interventions for patients with lymphatic disease can also be attempted. Thoracic duct embolization and selective lymphatic embolization have been used to successfully treat patients with lymphatic insufficiency (75–77). Our institution prefers to use selective lymphatic embolization as it leaves the thoracic duct intact for other possible interventions in the future (64). Further, thoracic duct embolization can actually worsen abdominal symptoms in patients with multicompartment lymphatic disease. If selective lymphatic embolization is not successful in these patients with multicompartment involvement, there has been early promise in transcatheter thoracic duct decompression, although this procedure requires considerable technical expertise (78). Finally, in atriopulmonary Fontan patients with persistent atrial arrhythmias contributing to heart failure symptoms, catheter ablation has been demonstrated to be safe and effective at reducing arrhythmia burden, although approximately half of patients will experience a documented episode of recurrent atrial arrhythmia post procedure and close to one third will require repeat ablation (79).

### Surgical interventions

Surgical interventions should also be considered in a patient experiencing Fontan circulatory failure. These include Fontan takedown, which is typically used in patients in the early postoperative period, Fontan revision, used in patients with Fontan obstruction, and Fontan conversion to a lateral tunnel or extracardiac Fontan, which is used in patients with an atriopulmonary type Fontan (80). Fontan takedown is accompanied by a very high early mortality, though it may be the only option for patients who are intolerant of Fontan physiology but may tolerate Glenn physiology until a transplant can be obtained. Fontan revision for surgical relief of Fontan obstruction can improve lymphatic dysfunction and Fontan conversion is often performed in conjunction with atrial arrhythmia surgery or pacemaker implantation to improve flow dynamics, lower incidence of arrhythmia and improve functional status (65, 81). The main drawback of surgical revision in this population is the high upfront risk. Fontan conversion has an early mortality at approximately 5%–10% with significant variability in outcomes among centers (81–83). In an analysis of the Society of Thoracic Surgeons Congenital Heart Surgery Database (STS-CHSD), Fontan revision had the highest reported mortality among all adult congenital cardiac operations (83). Risk factors for worse outcomes in patients undergoing Fontan revision include older age, presence of any STS-CHSD defined preoperative risk factor including preoperative mechanical circulatory support, diabetes, hepatic dysfunction, hypercoagulable state, renal dysfunction, complete heart block, pacemaker, implantable ICD and prior cardiothoracic operation, and concomitant pulmonary artery reconstruction (82).

Surgical innominate vein reimplantation to the lower-pressure pulmonary venous atrium (“turn down”) is another strategy that has been described in Fontan patients with lymphatic dysfunction, although it will worsen cyanosis (84, 85). Alternatively, in certain subsets of patients with a borderline second ventricle, biventricular conversion with possible recruitment of the systemic ventricle before conversion could be considered (86). Finally, in single ventricle patients with significant AV valve regurgitation, surgical intervention on the valve is an option. There is a movement in the field toward identifying the mechanism of AV valve insufficiency with earlier valve repair, perhaps prior to Fontan, and more aggressive valve replacement in specific situations (87–89). Although these procedures can be successfully performed, there remains a question of durability of repair (90).

### Medical treatments

Medical options include the use of many of the drugs used in biventricular heart failure, but with less evidence to support the practice. A recent systematic review of the evidence for medical therapy in prevention of Fontan circulatory failure identified only 9 studies which included only 267 Fontan patients total (91).

Diuretics are frequently used in systolic and diastolic dysfunction for symptomatic control. While they may reduce the filling pressure and improve symptoms of heart failure, they also decrease cardiac output and are often associated with electrolyte disarray and potential worsening of renal dysfunction (1). Reverse remodeling agents including renin-angiotensin-aldosterone system blockers and β-blockers are frequently used in clinical practice, albeit with little data to support the benefit of these drugs in single-ventricle patients with ventricular dysfunction or AV valve regurgitation (12, 45, 92–94). In one cohort study of patients after Fontan, 10 weeks of enalapril administration did not alter baseline hemodynamics, exercise capacity, or diastolic function and there was a suggestion of a worsening of short term exercise capacity (93). In another small cohort treated with ACE inhibitors for 6 months, no difference was seen in ventricular ejection fraction (94). Data demonstrating improved ventricular function, reversal in left-ventricular hypertrophy, and improved ventricular volume overload in patients with two-ventricle circulations with mitral or aortic valve regurgitation are often extrapolated to single ventricle patients, but there have been no studies specifically examining this effect in the patients with AV valve regurgitation and a Fontan (95–97). Use of beta blockers has also been examined in patients with a Fontan with heart failure, but a randomized control trial of beta blockade in children demonstrated that use of beta blockers may actually be harmful in patients with a systemic right ventricle (98). There is some evidence for a role for high dose spironolactone in failing Fontan patients specifically with PLE, although again the data remain limited to small series (65, 99, 100).

Modulators of pulmonary vascular resistance have also been used in this population, with some evidence supporting the practice. Phosphodiesterase type 5 inhibitors are of particular interest given their ability to decrease PVR, improve ventricular end-diastolic elastance, decrease arterial elastance, and increase ventricular end-systolic elastance, which in turn may improve preload and ventriculoarterial coupling (101). Small studies using sildenafil have demonstrated improved exercise capacity, improved exercise hemodynamics, and improved ventricular performance (102–106). Recent results from the larger FUEL trial demonstrated no significant change in peak oxygen consumption in patients with a Fontan circulation treated with udenafil. However, there was a statistically significant improvement in submaximal exercise measures including oxygen consumption, work rate, and ventilatory efficiency at the anaerobic threshold (107). Bosentan has also been examined in small scale trials and has shown mixed results in its effect on exercise capacity and New York Heart Association functional class (108–110). Finally, a single study of inhaled iloprost prior to exercise stress test demonstrated improved peak oxygen pulse and peak oxygen consumption and was particularly beneficial in patient with impaired exercise function at baseline (111). One subpopulation of patients with Fontan failure that may benefit from pulmonary vasodilation in particular are patients with lymphatic failure (65, 112).

If mediators of pulmonary vascular resistance are not effective, patients with protein losing enteropathy have several additional medical options which may be effective in some patients. Midodrine, an alpha-1-receptor agonist, has shown some promise, with a recent case series of four patients with refractory PLE demonstrating symptomatic improvement leading to deferral of transplant evaluation in two patients and transplant delisting in one patient (113). It is hypothesized that midodrine works by its effect on tone in the smooth muscle cells in the lymphatic vessels, though additional work is needed to confirm this hypothesis (113, 114). Additional medical treatments for patients with PLE include oral budesonide and octreotide, but use may not reduce the need for transplant over the long term (65, 115, 116).

Antiarrhythmics for control of atrial arrhythmias can improve systolic dysfunction and AV valve regurgitation. However, use of antiarrhythmic medications in patients with Fontan has been associated with a higher risk of recurrence and more adverse events compared to interventions such as catheter ablation or Fontan conversion (1, 117). In patients with a pacemaker, employing a pacing mode with atrioventricular synchrony has been demonstrated to improve Fontan hemodynamics (69). In Fontan patients with heart block or intact conduction with significant dyssynchrony, cardiac resynchronization therapy with dual-site ventricular pacing has shown mixed results though recent data has demonstrated improved indices of ventricular function that are less dependent upon ventricular anatomy and geometry. These improved indices also appear to correlate with improved New York Heart Association classification (118).

Finally, exercise training as therapy for Fontan patients should be considered, as it has been examined in a number of small studies with some demonstrating an increase in peak oxygen uptake, an increase in cardiac output, or an increase in quality of life with training (119). More study is needed to determine the optimal training type and program. However, if there is significant Fontan circulatory failure it is unlikely that exercise training alone will prevent the need for advanced heart failure therapies.

### Advanced heart failure therapies

#### Intravenous inotropes

In Fontan patients with low cardiac output and compromised end-organ perfusion, intravenous inotropic support may be necessary (6). There are few data regarding the use of inotropes in patients with a Fontan circulation, and thus inotropic support in these patients is typically based on anecdotal experience and extrapolation of data from adult and pediatric patients with a biventricular circulation (120–123). Pediatric heart failure guidelines recommend milrinone or dobutamine as first line therapy with the addition of epinephrine for refractory hypotension (6). In patients with PLE, dopamine therapy can be trialed, as there is evidence it may induce remission in patients with PLE possibly through effects on lymphatic receptors (124).

Despite the lack of data, the inotropic, lusitropic, and vasodilatory properties of phosphodiesterase-3 inhibitors such as milrinone make this class of drug appealing for treatment of the failing Fontan (125, 126). Phosphodiesterase-3 inhibitors have been demonstrated to improve early postoperative hemodynamics in Fontan patients by increasing cardiac index and stroke volume index but this effect has not been specifically examined in Fontan patients remote from surgical repair (127). Interestingly, the molecular myocardial effects from milrinone appear to be unique in patients with a failing single right ventricle as compared to pediatric patients with dilated cardiomyopathy (128).

The effect of chronic inotrope use in this population remains unknown and may be harmful (129). Therefore, we recommend chronic inotrope use only in palliative circumstances or as a bridge to transplant.

#### Mechanical circulatory support

Due to the increasing number of Fontan patients with circulatory failure and the long wait times for organ transplantation, the use of durable mechanical circulatory support (MCS) in this population is becoming increasingly common (130–135). Unfortunately, outcomes remain inferior when compared to patients with a biventricular circulation, though seem to be improving (130, 135–140). Encouragingly, the ACTION Network recently reported no deaths among 5 patients with a Fontan circulation supported with a Heartmate 3 device (139).

Despite improving outcomes, there remain several challenges to supporting patients with a Fontan circulation with MCS. Surgical challenges include a redo sternotomy, cumbersome mediastinal dissection and dense trabeculations within the ventricle which can require resection to avoid inflow obstruction, and challenging geometry of cannula position in single ventricle patients (134). Novel surgical approaches including atrial cannulation and excision of AV valve may be needed to place devices in smaller patients (141). Further, a ventricular assist device may not address the underlying cause of the Fontan failure. MCS is typically most beneficial in patients with ventricular dysfunction, but may not be as useful in patients with other forms of Fontan failure, where preload is limited and increasing the cardiac output can further worsen right-sided congestive symptoms (1, 134). In patients with Fontan circulatory failure without significant pump dysfunction, innovative support strategies may need to be employed. There have been single case reports of successful use of BiVAD, Syncardia Total Artificial Heart and single subpulmonary VAD in patients with Fontan circulations, but these strategies have not been widely adopted (142–144).

Additionally, temporary VAD support with Impella has been described in a limited number of pediatric and adult patients with a Fontan circulation with cardiogenic shock with encouraging results (145, 146). These devices may be used as a bridge to recovery or more durable support. Noted challenges in this population include abnormal relationships between the great arteries and abnormal orientation of the atrioventricular valves relative to the outflow tracts. Reported complications included one procedural death, increasing aortic valve insufficiency, access site thrombus, extremity ischemia, hemolysis, and renal failure.

#### Transplant

Transplant remains the definitive treatment for circulatory failure after Fontan; however, there is significant variability in access to transplant across centers (23). A recent analysis of the STS-CHSD demonstrated an increasing number of transplants performed in patients with a Fontan over the last decade, due primarily to an increase in patients with HLHS (82). Waitlist mortality is considerable, although pediatric patients with a Fontan circulation have demonstrated similar pretransplant survival (78% at 6 months and 74% at 12 months) to other patients listed for transplant both with and without CHD (147). However, adult patients with CHD listed 1A for transplant have higher wait list mortality than non-congenital heart patients, estimated at approximately 1 in 4 (148). Risk factors for death while waiting in the pediatric population include younger age, status 1 listing, shorter interval since Fontan and ventilator support (147).

Transplantation in patients with a Fontan is high-risk, but outcomes have improved in recent decades (29, 147, 149–152). Recent approximate 1 year survival was 89%. This 1 year survival is similar to non-Fontan CHD patients, although continues to lag behind outcomes in patients without congenital heart disease (147, 151). Operative mortality and composite major morbidity and mortality remain high at 7.6% and 35%, respectively (82). Fontan patients have several baseline risk factors including multiple prior cardiac surgeries with exposure to blood products and homograft and significant aortopulmonary collateral burden. Therefore, at transplant they are at increased risk of surgical complications including complicated dissection, bleeding, and longer ischemic time, as well as longer term complications such as rejection due to presensitization (153, 154). Identified risk factors for worse outcomes include increasing age among adult patients and preserved ventricular function (28, 82). This may be because Fontan patients with preserved ventricular function are more likely to have long term malnutrition, immunosuppression, and pulmonary complications, therefore there is possible benefit to earlier referral in this population. Patients with PLE who survive transplant have been shown to have resolution of their PLE (147, 150, 155). The most common causes of death after transplant in patients with a Fontan are similar to other transplant populations and include infection, early graft failure, rejection and sudden death (147).

One area of active research in Fontan management is in multiorgan transplant. Fontan associated liver disease is common and some patients with hepatic dysfunction will undergo heart-liver transplantation. There is discrepant data regarding whether outcomes are better in Fontan patients with cirrhosis undergoing heart-liver transplant vs. heart transplant alone (156–158). It is not known which patients are likely to have reversal of hepatic dysfunction after heart transplant alone and thus universal indications for heart-liver transplant remain undefined.

## Summary

Circulatory failure in patients with a Fontan circulation is becoming an increasingly common issue. Management of Fontan failure is dependent upon identification of the underlying etiology, which may include ventricular systolic and diastolic dysfunction, atrioventricular valve regurgitation, arrhythmia, and lymphatic insufficiency. Surgical and catheter-based interventions may be effective in addressing specific causes of Fontan failure but can be high risk for patients. Medical therapies are frequently tried but not very effective overall, and thus advanced heart failure therapies are often required, including inotropic infusions, ventricular assist devices, and heart transplantation. There remains considerable room for improvement in the management and outcomes of patients with circulatory failure after Fontan surgery.
